# Carvacrol Prodrugs with Antimicrobial Activity Loaded on Clay Nanocomposites

**DOI:** 10.3390/ma13071793

**Published:** 2020-04-10

**Authors:** Piera Eusepi, Lisa Marinelli, Fátima García-Villén, Ana Borrego-Sánchez, Ivana Cacciatore, Antonio Di Stefano, Cesar Viseras

**Affiliations:** 1 Department of Pharmacy, University “G. d’Annunzio” of Chieti-Pescara, Chieti, 66100 Abruzzo, Italy; piera.eusepi@unich.it (P.E.); ivana.cacciatore@unich.it (I.C.); adistefano@unich.it (A.D.S.); 2 Department of Pharmacy and Pharmaceutical Technology, School of Pharmacy, University of Granada, 18071 Granada, Spain; fgarvillen@ugr.es (F.G.-V.); anaborrego@iact.ugr-csic.es (A.B.-S.); cviseras@ugr.es (C.V.); 3 Andalusian Institute of Earth Science, CSIC-University of Granada, Armilla, 18100 Granada, Spain

**Keywords:** carvacrol, clay, montmorillonite, prodrugs

## Abstract

Background: Carvacrol, an essential oil with antimicrobial activity against a wide range of pathogens, and its water soluble carvacrol prodrugs (WSCP1-3) were intercalated into montmorillonite (VHS) interlayers to improve their stability in physiological media and promote their absorption in the intestine. Methods: Intercalation of prodrugs by cation exchange with montmorillonite interlayer counterions was verified by X-ray powder diffraction and confirmed by Fourier transform infrared spectroscopy and thermal analysis. Results: In vitro release studies demonstrated that montmorillonite successfully controlled the release of the adsorbed prodrugs and promoted their bioactivation only in the intestinal tract where carvacrol could develop its maximum antimicrobial activity. The amount of WSCP1, WSCP2, and WSCP3 released from VHS were 38%, 54%, and 45% at acid pH in 120 min, and 65%, 78%, and 44% at pH 6.8 in 240 min, respectively. Conclusions: The resultant hybrids successfully controlled conversion of the prodrugs to carvacrol, avoiding premature degradation of the drug.

## 1. Introduction

Antibiotics resistance is occurring due to use of antibiotics in prevention of microbial infections without previous antibiogram together with treatment noncompliance and routine application in agricultural and aquaculture [1]. The World Health Organization (WHO) estimated that about 700,000 people die every year because antibiotics have become ineffective against common pathogens, and that 10 million lives per year will be at risk by 2050 [2]. Research must be promoted to find novel classes of antibiotics with new action mechanisms. Carvacrol (2-methyl-5-isopropylphenol), a phenolic monoterpene abundant in essential oils of aromatic plants especially of Labiatae family (*Origanum*, *Satureja*, and *Thymus*), has shown antibacterial, antifungal, and antiviral properties against resistant pathogens [3,4]. The mechanism of action of carvacrol against microorganisms is associated with the presence of a hydroxyl group and an aromatic ring. The hydroxyl group generates a K^+^ efflux and H^+^ influx across the microbial membrane, generating an imbalance in osmotic pressure, inactivation of enzymes, alteration of pH gradient, change in membrane potential, and thus reduced ATP synthesis—factors that lead to microbial cell death [5,6]. Additionally, the aromatic group confers adequate lipophilicity (octanol/water partition coefficient (log*P* o/w) of 3.64) for carvacrol to accumulate into the cytoplasmic layer, thus distorting and destabilizing the membrane, which becomes more fluid and permeable to small molecules. These structural characteristics are also responsible for technological and biopharmaceutical deficiencies, such as low water solubility (0.11 mg/mL) and high lipophilicity [7,8]. When orally administered, carvacrol is mainly and rapidly adsorbed in the stomach [9,10], though small intestine absorption would lead to maximum antimicrobial activity [11].

Carvacrol prodrugs, with improved water solubility obtained by modification of the aromatic group with sulfonic moiety or by glycosylation and esterification of the monoterpenoid hydroxyl moiety with glycosyl group and sulphur derivatives, have been proposed. However, inclusion of organic moieties resulted in reduced biocompatibility [12,13]. The synthesis of biocompatible prodrugs with improved solubility, stability, and biopharmaceutical profiles is desirable [14].

**Figure 1:**
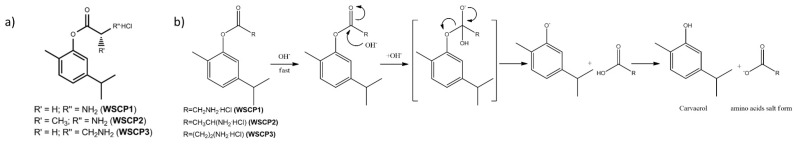
(**a**) Chemical structures of water soluble carvacrol prodrugs (WSCP1-3) and (**b**) proposed bioactivation of WSCP1-3 under alkaline conditions.

In a previous work, carvacrol was derivatized with natural amino acids (ionized glycine, alanine, and β-alanine) to obtain highly biocompatible prodrugs (Figure 1) [15]. The resultant prodrugs showed antimicrobial activities and increased water solubility in comparison to carvacrol. These prodrugs did not convert to carvacrol in the stomach, preventing premature absorption of the drug. Once the intestinal conditions were reached, they undergo a fast conversion to the parent drug. To maximize the antimicrobial activity of carvacrol, prodrugs conversion rates in upper intestinal conditions should be reduced. Drug delivery systems have been extensively investigated in biopharmaceutical applications as an alternative approach to counteract diseases difficult to conventionally manage [16,17]. In this field, clay minerals are natural inorganic excipients successfully used as drug carriers that are able to increase drug stability and solubility, as well as to modify the rate, time, or site of drug release and prevent or reduce side effects [18,19,20]. In particular, bentonite, included in the United States Pharmacopeia (USP) and in the European Pharmacopoeia (PhEur), is mainly constituted by montmorillonite, a smectite phyllosilicate with a 2:1 geometry in which the alumina sheets condense between two silica sheets in a linear way, forming a nanometric interlayer space able to retain drug molecules [21]. The ability to retain drugs into the lumen of the nanosized clay layers is due to isomorphic substitutions. The exchanges of Si^4+^ by Al^3+^ and Al^3+^ by Mg^2+^ in the silica tetrahedral sheet and alumina octahedral sheet, respectively, generate a net negative charge that is generally neutralized by adsorption of inorganic cations in the interlayer space (Na^+^, Ca^2+^, K^+^, and Mg^2+^). These inorganic cations can be replaced by organic cations, subsequently causing montmorillonite interlayer expansion ascribed to the bigger size of the organic molecules [22,23]. The combination of clay minerals with other organic molecules (either active or not) produce compounds commonly known as nanohybrids. Several organic/inorganic nanohybrid systems were prepared with several drugs, improving the stability and/or solubility of the encapsulated molecules [24,25,26].

Under these premises, in the present work, we intercalated three water soluble carvacrol prodrugs (WSCP1-3) (Figure 1a) into montmorillonite layers to protect them from premature enzymatic or chemical hydrolysis and to obtain a modified release of the carried compound in small intestine conditions to increase carvacrol microbial activity. Stability studies were performed before and after intercalation to evaluate the effective stabilization. Solid-state characterization and release studies were conducted to determine the potential use of the nanohybrids as drug delivery systems.

## 2. Materials and Methods

Three carvacrol prodrugs (WSCP1-3) were synthetized following Marinelli et al. [15]. Montmorillonite (VHS, HS grade, Veegum^©^) was purchased from Vanderbit Minerals, LLC, Normalk, CT, USA.

### 2.1. Chemical Conversion of Carvacrol Prodrugs

Prodrugs solutions (1 mg/mL) in water and buffer solutions (pH 1.2, 6.8, and 7.4) were incubated at 37 °C under magnetic agitation (650 rpm) to determine the WSCP1-3 conversion rates to carvacrol [27]. We removed 400 μL samples at various time intervals and drug concentrations were analyzed by high performance liquid chromatography (HPLC) using an Agilent 1260 Infinity II HPLC (Agilent, Santa Clara, CA, USA) consisting of quaternary pump, autosampler, column oven, and UV-Vis diode-array detector. Samples were run in an Agilent Poroshell 120 EC-C18 column (4 μm, 4.60 × 150 mm; Agilent, Santa Clara, CA, USA) maintained at 25 °C. The mobile phase consisted of a mixture 50:50 v/v H_2_O MilliQ (Merck KGaA, Darmstadt, Germany) and CH_3_CN with 0.1% trifluoracetic acid (TFA) flushing at an isocratic flow rate of 1 mL/min. The detector was set at a wavelength of 270 nm. Data were processed using LC Open LAB HPLC 1260 software (Agilent, Santa Clara, CA, USA). Results are reported as relative difference (RD%) of prodrugs conversion, calculated with Equation (1):

(1) $$\mathrm{RD}\%=\frac{C_{i}-{\;C}_{f}}{C_{i}}\;\times 100$$

where C_i_ is the prodrug concentration at zero time point and C_f_ is the prodrug concentration at the end of incubation [28]. A linear calibration curve with a coefficient of determination (*R*^2^) value of 0.9999 was obtained by analyzing 20 μL of WSCP1-3 solutions at the range of 5–500 μg/mL in the HPLC system.

### 2.2. Preparation of WSCPs/VHS Hybrids

WSCP1-3-clay hybrids were obtained by suspending VHS into the corresponding WSCP1-3 aqueous solutions in a 1:1 ratio w/w. Suspensions were shaken for 24 h in a water bath at 22 °C [28,29]. After 24 h, suspensions were centrifuged at 10,000 rpm (30 min) and the supernatant was recovered and filtered through 0.45 μm Millipore^©^ membrane (Merck KGaA, Darmstadt, Germany). The amount of adsorbed prodrugs (q_e_, mg/mg) was calculated according to Equation (2):

(2) $$q_{e}=(C_{0}-C_{e})\;\times \;V/W$$

where *C*_0_ and *C*_e_ are the initial and the equilibrium supernatant concentration (mg/mL) determined by HPLC analysis, respectively, *V* is the volume of the suspensions (mL), and *W* is the mass of suspended VHS (mg). The nanohybrids were stored in a desiccator at room temperature for future analysis [28,29].

### 2.3. Solid State Characterization

#### 2.3.1. X-ray Power Diffraction (XRPD)

XRPD was conducted using a Philips^®^ X-Pert diffractometer (X’Pert^3^ MRD, Malvern, Cambridge, UK) with CuKα radiation. The diffractograms were analyzed using XPOWDER^®^ software (XPowder12, ver 2012.01.01, Granada, Spain).

#### 2.3.2. Fourier-Transform Infrared Spectroscopy (FTIR)

FTIR spectra were recorded on an infrared spectrophotometer (JASCO 6200, JASCO International Co., Easton, MD, USA), equipped with SPECTRA MANAGER v2 software (JASCO, International Co., Easton, MD, USA) and with an attenuated total reflectance (ATR) accessory. FTIR measurements were performed from 400 to 4000 cm^−1^ with a 0.25 cm^−1^ resolution.

#### 2.3.3. Thermal Analysis

Thermogravimetric analysis (TGA) and differential scanning calorimetry (DSC) were conducted using a METTLER TOLEDO mod (Mettler Toledo, Columbus, OH, USA). The TGA/DSC1 calorimeter was coupled with an FRS5 sensor and a microbalance (precision 0.1 μg; Mettler-Toledo GMBH, Mettler Toledo, Columbus, OH, USA). Analyses were performed in air atmosphere with a heating rate of 10 °C/min in the temperature ranges of 30–950 °C for TGA and 30–400 °C for DSC.

#### 2.3.4. In Vitro Release Studies

Release studies were conducted in a dissolution tester (Sotax AT7, SOTAX Group, Aesch, Switzerland). A total of 100 mg of WSCP1-3 loaded-VHS were dispersed in 250 mL of 0.1 M HCl and pH 6.8 phosphate buffer solutions, simulating the gastric and intestinal fluids without enzymes, respectively. During the test, temperature was maintained constant at 37 °C and agitation was achieved using a paddle system set to 100 rpm. At predetermined time intervals, 5 mL of dissolution medium was withdrawn, filtered with 0.45 μm Millipore^©^ membranes, and replaced with an equal volume of the corresponding fresh dissolution medium. Concentrations of the corresponding prodrugs and carvacrol were simultaneously measured in withdrawn aliquots by HPLC using the following equation [30,31]:

(3) $$Q_{t}=V_{m}C_{t}+\overset{t-1}{\underset{t=0}{\sum }}V_{a}C_{i}$$

where *V_m_* and *C_t_* are the volume and the concentration of drugs at time *t*, respectively; V*_a_* is the volume picked up; and *C_i_* is the concentration of drug at time *i*.

### 2.4. Statistical Analysis

The results are presented as the mean ± standard deviation of three experiments. GraphPad Prism Software, version 7 (GraphPad Software, La Jolla, CA, USA) and Microsoft Office 365 ProPlus, version 1908 were used to obtain graphs and statistics.

## 3. Results and Discussion

### 3.1. Chemical Conversion of Carvacrol Prodrugs

**Figure 2:**
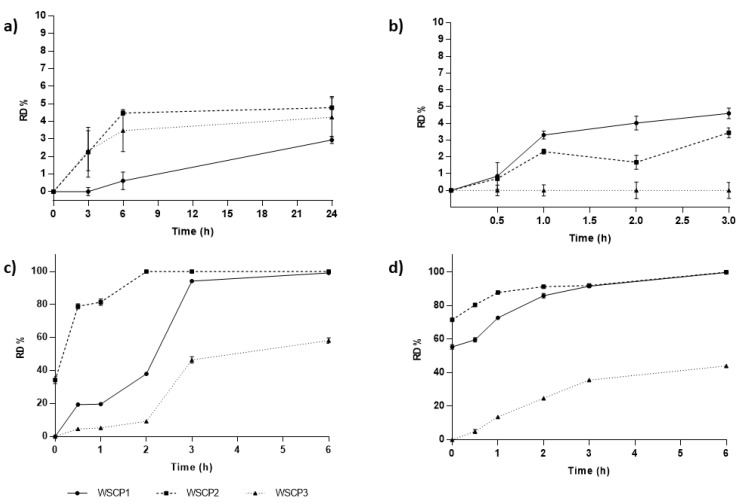
Relative difference (RD%) of prodrugs conversion over time in (**a**) water and different buffer media at pH (**b**) 1.2, (**c**) 6.8, and (**d**) 7.4. Values are the means of three experiments and error bars represent the standard deviation.

Preliminary in vitro studies were performed on free WSCP1-3 to evaluate their chemical stability under physiological conditions mimicking the gastric and intestinal environmental. Cumulative amounts of carvacrol converted from the prodrugs over time are presented in Figure 2. All the studied prodrugs showed high water stability (<5% hydrolyzed at 24 h). Under fasting conditions, pH values range from 1.4 to 2.1 in the stomach, 4.9 to 6.4 in the duodenum, 4.4 to 6.6 in the jejunum, and 6.5 to 7.4 in the ileum [32]. Variable transit rates may determine the degree of prodrug conversion. Gastric emptying times in fasted conditions are usually assumed to be around 60 minutes, but they may even reach up to two or three hours [33]. According to the results, all the studied prodrugs successfully controlled premature conversion to carvacrol at pH 1.2, being lower than 5% even after 180 min (Figure 2b). As previously described, an ideal carvacrol prodrug should be stable in the stomach but convert to the drug in intestinal conditions [14]. To determine the ability of the studied prodrugs to convert to carvacrol in intestinal conditions, hydrolysis values at pH 6.8 and 7.4 were also determined (Figure 2c,d). Intestinal transit rates are much more predictable and constant than gastric transit. The intestinal transit time of medicinal products is reported to be about three hours [34].

Highest conversion rates were measured both at pH 6.8 and 7.4. Prodrugs were particularly labile at intestinal pH, suggesting that after oral administration, the prodrugs pass through the stomach unmodified and rapidly transform into carvacrol in the intestinal tract, where the drug can be adsorbed [35]. The biggest advantage of the proposed strategy is the possibility of fine-tuning the conversion degree and the site where it will occur by selecting the structure of the pro-moieties. The pseudo-first-order dissociation rate constants (K_obs_) and half-live times (t_1/2_) of the prodrugs were determined by plotting the logarithm of WSCPs concentration at various time intervals (Table 1). The nature of the amino acid esters affected the rate of conversion. We observed that the alanine derivative underwent a faster hydrolysis than glycine and β-alanine prodrugs, as demonstrated by their t_1/2_ values. Both kinetic constants and half-lives of the studied prodrugs might change in vivo, where esterases and other enzymes affect the values.

The proposed mechanism of prodrugs bioactivation consists of the base-catalyzed hydrolysis of the ester group that faster led to the release of carvacrol and the amino acidic salt form (Figure 1b). In particular, the reaction occurs at the electrophilic carbonyl carbon, where the nucleophilic OH^−^ attacks the carbonyl carbon to form a tetrahedral intermediate that subsequently undergoes acyl cleavage with liberation of the phenolate anion and the amino acid. Finally, the amino acid transfers its proton with subsequent formation of carvacrol and the anion form of the amino acids. The base-catalyzed reaction is irreversible since the final proton transfer from the amino acids to the phenolate anion is the prevalent reaction; it is difficult for the hydroxyl anion to attach the anion form of the amino acids due to electrostatic repulsion [36].

### 3.2. Solid State Characterization of Drug–Clay Hybrids

#### 3.2.1. Adsorption Equilibrium Studies

**Figure 3:**
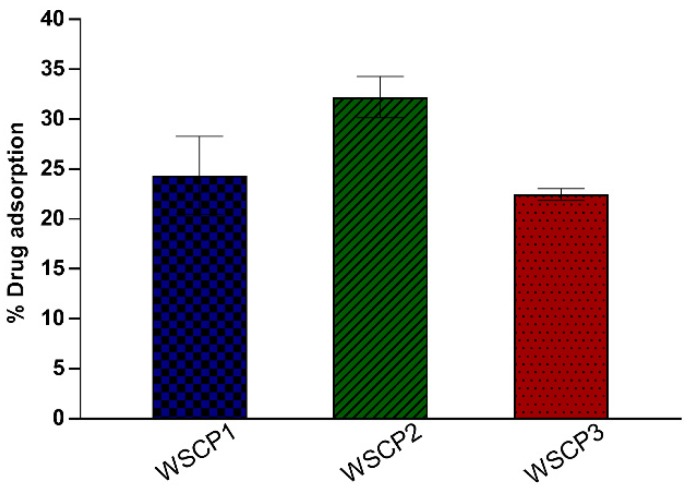
Percentage WSCP1-3 adsorption into Montmorillonite (VHS). Each experiment was repeated three times and error bars represent the standard deviation.

The amount of carvacrol prodrugs retained by VHS were 24%, 32%, and 22% w/w for WSCP1, WSCP2, and WSCP3, respectively (Figure 3). These findings are attributed to the extensive internal and external surfaces and to the high cation exchange capacity of the VHS [37]. These values are similar to those previously obtained with the same clay and different drugs [38].

#### 3.2.2. XRPD

**Figure 4:**
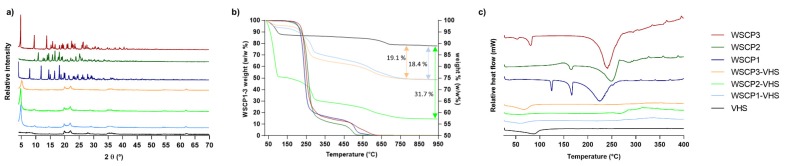
Nanohybrids and raw components analyzed by (**a**) X-ray Power Diffraction (XRPD), (**b**) thermogravimetric analysis (TGA), and (**c**) differential scanning calorimetry (DSC).

To provide information related to the crystal structure of WSCP1-3, VHS and nanohybrids, XRPD pattern was carried out. As shown in Figure 4a, the X-ray pattern of carvacrol derivatives was typical of a crystalline substance, exhibiting a series of sharp crystallinity peaks, with the most intense ones at 18.2°, 11.8°, and 7.9° 2θ for WSCP1; 16.7°, 10.9°, and 18.1° 2θ for WSCP2; and 4.8°, 9.5°, and 13.8° 2θ for WSCP3. In contrast, VHS showed a typical diffraction pattern of montmorillonite, with a characteristic peak at 7.4° 2θ corresponding to an interlayer distance (d_001_) of 11.5 Å [39,40]. In hybrids samples, the characteristic crystalline signals of pure WSCP1–3 disappeared. This could be ascribed both to a successful interaction between VHS and corresponding prodrugs [41] as well as to amorphization of the prodrugs, which could also explain the absence of crystal reflections. The effective inclusion of drug molecules within the VHS interlayer space was confirmed by the shift in the d_001_ peaks to lower values. Specifically, the corresponding interlayer spaces of VHS changed from 11.5 Å (unloaded VHS) to 17.70, 18.00, and 16.40 Å in WSCP1-VHS, WSCP2-VHS, and WSCP3-VHS, respectively.

According to these results, the presumed mechanisms responsible for drug/VHS interaction were the adsorption onto the free surface of laminar clay due to Van der Waals forces and replacement of interlayer cations with protonated prodrug through cation exchange [18,42].

### 3.3. TGA and DSC

DSC and TGA were performed to compare the behaviour of loaded nanohybrids to that of the individual components. These thermal analyses allowed us to highlight any significant enthalpy and masses loss changes within the investigated temperature interval.

The thermal profiles of pure WSCP1-3, VHS, and the corresponding hybrids are compared in Figure 4b,c. A summary of the thermal events together with their corresponding weight losses is provided in Table 2. TGA of raw VHS showed desorption of hydration water from 40 to 120 °C, which corresponded to the endothermal event at 87 °C of the DSC [43]. The second weight loss of 4.3% w/w occurred from 600 to 870 °C and belonged to dehydroxylation of structural –OH from VHS sheets [44,45]. None of the prodrugs (WSCP1-3) showed mass losses before 200 °C (Figure 4b), though DSC analysis showed endothermic events for three of them before this temperature (Figure 4c), thus indicating thermal transitions. In particular, the lower endothermic peaks at 125 and 53 °C observed in WSCP1 and WSCP3, respectively, are associated with solid–solid phase transition, indicating the possibility of polymorphism [46,47,48]. The peaks at 166 °C in WSCP1, 165 °C in WSCP2, and 81° C in WSCP3 with enthalpy (ΔH) of −47.17, −14.71, and −34.63 Jg^−1^, respectively, are due to melting transition of drug crystals. The TGA thermogram showed two degradation steps that belonged to thermolysis of the ester group with the subsequent evaporation of the carvacrol moiety and oxidation of the aminoacidic portion, respectively (Table 2). These events were detected in DSC (Figure 4c) in the form of large endothermic peaks at 224 °C with ΔH of −397.26 Jg^−1^, 248 °C with ΔH of −433.06 Jg^−1^, and 241 °C with ΔH = −465.11 Jg^−1^ for WSCP1, WSCP2, and WSCP3, respectively [47,48,49,50].

In the TGA of loaded hybrids WSCP1-VHS, WSCP2-VHS, and WSCP3-VHS, four mass losses were distinguished. The water loss event was more significant for the WSCP2-VHS hybrid (Figure 4b,c), whereas WSCP1-VHS and WSCP3-VHS underwent minimum weight losses (Table 2). In DSC, the melting and degradation peaks related to drugs in WSCP1-VHS and WSCP3-VHS completely disappeared and was very low intense in WSCP2-VHS, and shifted to a higher temperature. These results confirmed that carvacrol derivatives were intercalated into the interlayer spaces, producing major thermal stability due to the protection of clay minerals [31]. Given the TGA results, it is possible to estimate the drug loading of each hybrid: these results are in agreement with drug adsorption as reported in Figure 3 and Table 2.

### 3.4. Fourier Transform Infrared (FTIR)

**Figure 5:**
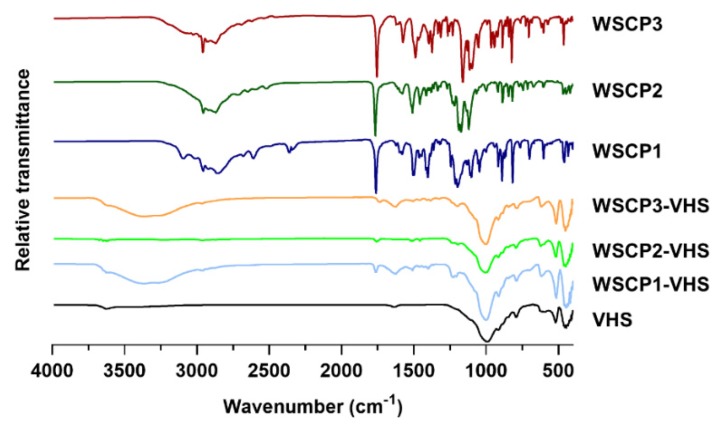
Nanohybrids and raw components analyzed by Fourier transform infrared (FTIR).

To elucidate the nature of interactions involved in the adsorption process FTIR spectra of WSCP1-3, VHS and drug-clay hybrids were recorded. Figure 5 represents the FTIR spectra of VHS, WSCP1-3, and drug-loaded hybrids. VHS showed the characteristics adsorption bands at 3620 and 914 cm^−1^ due to –OH stretching and bending vibrations of water bonded to the Si–O surface, respectively. The peaks at 3440 and 1636 cm^−1^ are associated, respectively, to –OH stretching and bending vibrations of physically adsorbed water. The band at 991 cm^−1^ is attributed to Si–O stretching vibrations of the layered silicate. The bands at 518 and 453 cm^−1^ are characteristics of Si–O–Si and Si–O–Al bending vibrations of the tetrahedral sheet, respectively, whereas peaks at 849 and 791 cm^−1^ are related to the stretching of Fe–Al–OH and Mg–Fe–OH bonds of the octahedral sheet, respectively [51,52,53]. Pure carvacrol derivatives showed typical bands of the carvacrol portion, such as the CH symmetric and asymmetric stretching between 2958 and 2861 cm^−1^, the isopropyl symmetric and asymmetric bending at 1383–1373 and 1361 cm^−1^, and the C=C stretching vibrations at 1622–1445 cm^−1^ [54]. Additionally, the bands that demonstrated the effective coupling reaction with the amino acids with subsequent formation of an ester group were the C=O stretching vibration at 1761–1755 cm^−1^ and the CC=O-O symmetric stretching at 1269–1188 cm^−1^ [55,56]. All these bands, though with lower intensity, were present in the three hybrids samples (WSCP1-VHS, WSCP-2-VHS, and WSCP3-VHS), suggesting that active molecules are adsorbed on mineral clay surfaces without any destruction of functional groups [57]. The characteristic WSCP1–3 bands also slightly shifted. The carbonyl stretching vibration changed from 1761–1755 cm^−1^ in pristine WSCP1–3 to 1734–1756 cm^−1^ in the corresponding hybrids. The C–O–C symmetric stretching was also altered from 1269–1206 cm^−1^ in prodrugs to 1205–1996 cm^−1^ in the hybrids. The shifting to lower wavenumber indicates that hydrogen bonding and Van der Waals forces are mainly responsible for the adsorption process [58], thus confirming the previously mentioned hypothesis about drug/clay interactions (see Section 3.2.2).

### 3.5. In Vitro Release Studies

**Figure 6:**
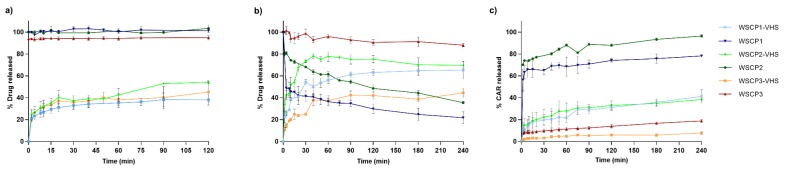
Amount of released WSCP1-3 at pH (**a**) 1.2 and (**b**) 6.8. Amount of carvacrol (CAR) released from WSCP1–3 and WSCP1–3 adsorbed–VHS hybrids at pH 6.8 (**c**). Each experiment was repeated three times and error bars represent the standard deviation.

In vitro studies on WSCPs-VHS systems were performed to define how nanocomposite can affect the WSCPs release over time guarantying their efficacious delivery and bioconversion in the target site. The drug release patterns of prodrugs from nanohybrids in comparison with pure WSCP1–3 in different buffer solutions at pH 1.2 and 6.8 mimicking, respectively, the gastric and intestinal environmental are reported in Figure 6a,b. We observed that the rate of drug release was dependent on pH, being higher at pH 6.8. Within 120 min at acid pH, the amount of the prodrugs released from VHS was 38%, 54%, and 45% for WSCP1, WSCP2, and WSCP3, respectively. At pH 6.8, 65% of WSCP1, 78% of WSCP2, and 44% of WSCP3 were released in 240 min. The release process can be explained by ion exchange reactions between the adsorbed prodrugs and the ions of the buffer medium [59]. In all cases, two-phase release was detected: a fast one associated with the desorption of prodrugs adsorbed on the VHS external surface, and a slower one due to the release of the intercalated molecules [30]. However, the percentage released did not reach 100% probably because the desorption reaction is an equilibrium process and ion exchange does not occur on all prodrugs molecules [39]. In comparison with the free WSCP1–3, the release was successfully retained in the nanohybrids as well as the prodrugs bioactivation. WSCP1–3 hydrolysis in the nanohybrid systems at acid pH did not occur (data not shown), limiting carvacrol gastric adsorption; whereas at pH 6.8, it occurred with increasing carvacrol adsorption in the small intestine (Figure 6c). Zhang et al. formulated alginate–whey protein microcapsules containing carvacrol that were able to deliver and release the monoterpene especially in the distal small intestinal tract. In particular, after oral administration in piglets, 34.1% and 3.5% of carvacrol were detected in the jejunum and ileum, respectively, at five hours after feeding. With increasing the size of microparticles, the recovery was enhanced to 32%, 50%, and 80% in the duodenum, jejunum, and ileum, respectively, at five hours after feeding, indicating the maximum adsorption during the distal small intestine [60].

## 4. Conclusions

Adsorption of carvacrol prodrugs onto pharmaceutical-grade VHS was achieved as confirmed by TGA, DSC, FTIR, and XRD analysis. In vitro desorption experiments and stability studies on the VHS nanohybrids revealed that the VHS structure was able to control WSCP1–3 release and protect the prodrugs from early hydrolysis in simulated gastrointestinal fluids. WSCPs loaded-VHS systems can be used to optimize carvacrol absorption in the small intestine to maximize its antimicrobial activity. Further experiments will be performed using an in vitro model to evaluate the antimicrobial activity of the investigated formulations against selected gastrointestinal bacteria strains.

## Figures and Tables

**Table 1:** The first-order dissociation rate constants (K_obs_) and half-live times (t_1/2_) of WSCP1-3 at pH 6.8 and 7.4 buffers.

pH	t_1/2_/K_obs_	WSCP1	WSCP2	WSCP3
pH 6.8	t_1/2_ (h)	2.33 (±0.07)	0.76 (±0.01)	18.17 (±0.10)
	K_obs_ (h^−1^)	0.2982 (±0.0106)	0.9027 (±0.0042)	0.0381 (±0.0002)
pH 7.4	t_1/2_ (h)	4.37 (±0.37)	0.88 (±0.07)	16.66 (±0.23)
	K_obs_ (h^−1^)	0.1937 (±0.0671)	0.7878 (±0.0629)	0.0420 (±0.0006)

Values are means of three experiments and standard deviation is given in parentheses.

**Table 2:** Summary of thermal events obtained from TGA and DSC analyses.

Sample	Temperature (°C) Range of Each Step	DSC Event (°C)	TGA Weight Loss (% w/w)	Thermal Event
VHS	40–120	87 (endo)	6.7	Desorption of hydration water
	600–750	-	4.3	VHS dehydroxylation
WSCP1	-	125	0.0	Solid–solid phase transition (polymorphism)
	-	166	0.0	Crystal melting point
	170–350	224	~80.0	Thermolysis of the ester group, subsequent evaporation of carvacrol moiety
	450–600	-	~15.0	Oxidation of the aminoacidic portion
WSCP2	-	165	0.0	Crystal melting point
	170–350	248	~90	Thermolysis of the ester group, subsequent evaporation of carvacrol moiety
	450–600		~10	Oxidation of the aminoacidic portion
WSCP-3	-	53	0.0	Solid–solid phase transition (polymorphism)
	-	81	0.0	Crystal melting point
	170–350	241	~80.0	Thermolysis of the ester group, subsequent evaporation of carvacrol moiety
	450–600	-	~15.0	Oxidation of the aminoacidic portion
WSCP1-VHS	40–100	60	3.3	Evaporation of hydration water
	170-–350	-	13.5	Drug decomposition
	500–750	-	7.2	Overlapped aminoacidic drug portion oxidation and VHS dehydroxylation
WSCP2-VHS	40–100	-	~50	Evaporation of hydration water
	170–350	267	~15	Drug decomposition
	500–750	-	5.7	Overlapped aminoacidic drug portion oxidation and VHS dehydroxylation
WSCP3-VHS	40–100	68	2.3	Evaporation of hydration water
	170–350	-	16.1	Drug decomposition
	500–750	-	5.2	Overlapped aminoacidic drug portion oxidation and VHS dehydroxylation
